# Total hospital stay for hip fracture: measuring the variations due to pre-fracture residence, rehabilitation, complications and comorbidities

**DOI:** 10.1186/s12913-015-0697-3

**Published:** 2015-01-22

**Authors:** Anthony W Ireland, Patrick J Kelly, Robert G Cumming

**Affiliations:** Department of Veterans’ Affairs, 300 Elizabeth St, Sydney 2000, New South Wales Sydney, Australia; School of Public Health, Edward Ford Building, University of Sydney 2006, New South Wales Sydney, Australia

**Keywords:** Hip fracture, Length of stay, Complications, Residential aged care, Rehabilitation

## Abstract

**Background:**

Hospital treatment for hip fracture is complex, often involving sequential episodes for acute orthopaedics, rehabilitation and care of contingent conditions. Most reports of hospital length of stay (LOS) address only the acute phase of care. This study identifies the frequency and mean duration of the component episodes within total hospital stay, and measures the impacts of patient-level and clinical service variables upon both acute phase and total LOS.

**Methods:**

Administrative datasets for 2552 subjects hospitalised between 1 July 2008 and 30 June 2009 were linked. Associations between LOS, pre-fracture accommodation status, age, sex, fracture type, hospital separation codes, selected comorbidities and complications were examined in regression models for acute phase and total LOS for patients from residential aged care (RAC) and from the community.

**Results:**

Mean total LOS was 30.8 days, with 43 per cent attributable to acute fracture management, 37 per cent to rehabilitation and 20 per cent to management of contingent conditions. Community patients had unadjusted total LOS of 35.4 days compared with 18.8 days for RAC patients (*p* <0.001). The proportion of transfers into rehabilitation (57 per cent vs 17 per cent, *p* <0.001) was the major determinant for this difference. In multivariate analyses, new RAC placement, discharge to other facilities, and complications of pressure ulcer, urinary or surgical site infections increased LOS by at least four days in one or more phases of hospital stay.

**Conclusion:**

Pre-fracture residence, selection for rehabilitation, discharge destination and specific complications are key determinants for acute phase and total LOS. Calculating the dimensions of specific determinants for LOS may identify potential efficiencies from targeted interventions such as orthogeriatric care models.

## Background

The hospital treatment of hip fractures is a complex process involving multiple services [1,2]. Following initial assessment, acute phase treatment is usually surgical, sometimes in a different hospital. Definitive discharge from the acute unit to the patient’s previous accommodation is the exception [2-4]. Transfer to another service for rehabilitation occurs in almost half of all cases [4,5] and transfers between hospital units for other reasons are not uncommon [4,6].

The traditional pattern of acute orthopaedic care followed by selective referral to rehabilitation or other aftercare is now frequently replaced by a variety of shared care models, with involvement of specialist geriatric and/or rehabilitation teams in the acute phase, or accelerated transit from the surgical ward to rehabilitation services [3,7,8]. Despite these developments, most reports of hospital stay for hip fracture describe only the acute surgical phase of treatment. This phase has a wide range of reported LOS from two days to more than two weeks [8,9]. In the few studies which report total LOS, mean values lie between 17 days and six weeks [3,9-11]. Total LOS for the current study has been previously reported at 30.8 days [4].

A wide variety of factors, including patient age [12], fracture type [3], preoperative delay [13] and specific comorbid conditions and complications [14] have been shown to impact the length of either acute phase or total LOS. However, the actual increase or decrease in LOS attributable to patient-level factors is rarely calculated, and then only for the acute phase of care [14].

The significance of residence in aged care institutions for risk factors and outcomes of hip fracture has been well described [15,16]. Less well documented is the impact of prior living status upon the duration and composition of hospital stay.

This study has two aims. First, to identify the proportion of total hospital stay due to acute phase treatment, rehabilitation and the management of contingent problems. Second, to identify and quantify the patient-related and clinical service factors associated acute phase and total LOS. For both aims, pre-fracture residential status is a major consideration.

## Methods

Episode-based datasets were obtained from the Australian Department of Veterans’ Affairs (DVA) for all veterans and war widows hospitalised for hip fracture (ICD-10-AM S72.0-S72.2) between 1 July 2008 and 30 June 2009. The Unique Identification Number (UIN) attached to every DVA record permitted linkage of continuous hospital episodes for individual patients, as well as linkage with RAC datasets and mortality records. Additional details of the of the data linkage process have been described previously [4].

Subjects were identified as community-dwelling or as residents of RAC facilities at the time of fracture and hospital admission. In Australia, defined reductions in capacity for activities of daily living and/or cognitive functionality, are statutory criteria for admission to RAC facilities, which include nursing homes [17].

### Data collection and classification

Hospital episodes contributing to total LOS were classified into three components - acute, rehabilitation and “other”. The acute phase included all episodes continuous with the index admission date with a primary diagnosis of hip fracture (ICD10-AM: S72.0-S72.2). The rehabilitation phase was the sum of all episodes coded (Z50.8-Z50.9) which were part of a continuous sequence of episodes following the index episode. The third component included all other episodes which were likewise in a continuous sequence following the index episode. These included care for comorbidities and complications or hospital time awaiting placement elsewhere. Every patient had an acute phase, but may or may not have had a rehabilitation or other phase.

The following variables were included in the dataset: pre-admission residential status (RAC or community), age, sex, fracture type, separation status (for each phase), clinical services (rehabilitation, intensive care, surgery), comorbidity and complications. Fracture type was classified as cervical (S72.01-72.04), trochanteric (S72.05, S72.10-72.11), subtrochanteric (S72.2) and ‘other’ (S72.00, S72.08). The dataset also included the comorbidities listed in the Charlson Index as modified for ICD-10-AM [18]. This information was extracted from all hospital episodes in the study year, up to and including the episode (s) comprising total LOS for the index hip fracture. Complications of skin ulceration (L89, L97), delirium (F05), anaemia (D62, D64.9), and urinary (N39), lower respiratory (J13-J15, J18, J20-22) and surgical wound (T81.4, T84.5-7) infections were also identified, due to associations with either LOS or unwanted outcomes following hip fracture [2,13,19]. Complications were identified only from those episodes comprising total LOS for the index fracture.

Hospital separation Code 9 - “separation to usual residence” or “other”- was interpreted as transfer to RAC if the patient had been in such care immediately prior to the index hospital admission. If hospital discharge and subsequent RAC admission dates were continuous, then transfer was also assumed regardless of the separation code. Details of the level of care provided within RAC for a given patient were not consistently available and were not analysed.

Since patients admitted from RAC or similar forms of supported living have different hospital trajectories from those who admitted from the community [12,20,21] data were tabulated and analysed separately for these two groups.

### Statistical analyses

Student’s t-test and Pearson’s Chi-squared test were used to assess differences in groups for continuous and categorical outcomes respectively. Total LOS, acute phase, rehabilitation phase and other phase LOS were tabulated for both RAC and community patients. Negative binomial regression models were then used to identify variables which significantly altered the length of acute phase and total LOS. Variables entered the model if univariate P <0.25 and, using backward elimination, remained in the final model if P < 0.05. For each variable in a final model, the average number of days greater or less than the baseline value (mean LOS when all predictor variables are zero or the referent group within a class variable) was calculated. All analyses were performed using SAS 9.3 (SAS Institute Inc; Cary, NC) or Excel 2010 (Microsoft Corporation, Redmond, WA).

Ethics approval was granted by the DVA Ethics Committee in December 2010.

## Results

There were 2552 patients hospitalised for hip fracture between 1 July 2008 and 30 June 2009. Linkage with RAC databases identified 27.7 per cent of patients as aged care residents at the time of hospital admission. Table 1 summarises the patient characteristics of the two sub-populations. There was a higher proportion of RAC patients aged 90 years or older (37 per cent vs 22 per cent, *p* < 0.001). The proportions of females, the distributions of fracture types and the proportions treated surgically were not significantly different. A greater proportion of community patients was admitted to Intensive Care (7.4 per cent vs 5.1 per cent, *p* = 0.035). Comorbidities and complications were similarly distributed apart from dementia (43.1 per cent vs 14.6 per cent, *p* < 0.001) and respiratory infection (12.3 per cent vs 9.1 per cent, P = 0.015). The proportion of transfers to rehabilitation was more than three times greater among community patients (57 per cent vs 17 per cent, *p* < 0.001).

**Table 1:** **Characteristics of study cohort by pre-fracture residential status**

	**Community patients (N = 1844)**		**RAC patients (N = 708)**		**All patients (N = 2552)**	
	**N**	**%**	**N**	**%**	**N**	**%**
Females	1148	62.3	444	62.7	1592	62.4
Age group						
<80	138	7.5	20	2.8	158	6.2
80-84	465	25.2	141	19.9	606	23.7
85-89	832	45.1	282	39.8	1114	43.7
90 +	409	22.2	265	37.4	674	26.8
Fracture type						
Cervical	712	38.6	269	38.0	981	38.4
Trochanteric	781	42.4	300	42.4	1081	42.4
Subtrochanteric	86	4.7	24	3.4	110	4.3
Other, unspecified	265	14.4	115	16.2	380	14.9
Rehabilitation	1050	56.9	122	17.2	1172	45.9
Surgical treatment	1543	83.7	611	86.3	2154	84.2
Intensive care	137	7.4	36	5.1	173	6.8
Comorbidities						
Dementia	269	14.6	305	43.1	574	22.5
Renal failure	253	13.7	96	13.6	349	13.7
Cardiac failure	231	12.5	104	14.7	335	13.1
Cardiac ischaemia	191	10.4	70	9.9	261	10.2
Diabetes	178	9.7	69	9.7	247	9.7
Respiratory disease	156	8.5	60	8.5	216	8.5
Stroke	108	5.9	53	7.5	161	6.3
Malignancy	131	7.1	30	4.2	161	6.3
Parkinson’s Disease	48	2.6	26	3.7	74	2.9
Complications						
Urinary infection	315	17.1	119	16.8	434	17.0
Skin ulceration	268	14.5	99	14.0	367	14.4
Anaemia	253	13.7	113	16.0	366	14.3
Chest infection	167	9.1	87	12.3	254	10.0
Delirium	166	9.0	80	11.3	246	9.6
Wound infection	49	2.7	14	2.0	63	2.5
Separation						
Private dwelling	1106	60.0	14	2.0	1120	44.7
RAC	391	21.2	575	81.2	966	37.1
Hospital, other	71	9.3	12	1.7	183	7.0
Death	176	9.5	107	15.1	283	11.2

### Components of LOS

For the total study population, 43 per cent of total LOS was attributable to acute fracture management, 37 per cent to rehabilitation and 20 per cent to other causes. Mean LOS values for the various components are shown in Table 2. There were 29012 hospital days for rehabilitation (1172 patients) and 15415 hospital days for “other” episodes (652 patients) out of the grand total of 78592 days.

**Table 2:** **Unadjusted mean values for components of total LOS for hip fracture**

	**Community patients**		**RAC patients**		**All patients**	
**Phase**	**N**	**Mean LOS (days)**	**N**	**Mean LOS (days)**	**N**	**Mean LOS (days)**
**All admissions**						
Acute	1844	14.1	708	11.6	2552	13.4
Rehabilitation	1050	25.1*	122	21.3	1172	24.8
Other	519	24.9*	133	18.6	652	23.6
Combined phases	1844	35.4*	708	18.8	2552	30.8
**Admissions which include rehabilitation**						
Acute	1050	11.8	122	10.9	1172	11.7
Rehabilitation	1050	25.1	122	21.3	172	24.8
Other	272	20.8	37	16.3	309	20.2
Combined phases	1050	42.3	122	37.2	1172	41.7
**Admissions without rehabilitation**						
Acute	794	17.0	586	11.8	1380	14.8
Other	247	29.5	96	19.4	343	26.7
Combined phases	794	26.2	586	14.9	1380	21.4

*Mean LOS for combined phases (total LOS) = weighted average from each component. For community patients = ((1844 × 14.1) + 1050 × 25.1) + (519 × 24.9))/1844 = 35.4 days.

The acute phase of care was significantly longer (14.1 days vs 11.6 days, *p* < 0.001) for community patients than for RAC patients. Both the proportion of patients transferred to rehabilitation and the total time in rehabilitation phase (25.1 vs 21.3 days) were significantly higher for community patients. The resulting per capita contribution to total LOS was (1050* 25.1/1844) =14.3 days for community patients and (122*21.3/708) = 3.7 days for patients from RAC (data in Table 2).

Linked hospital episodes attributed to neither acute fracture care nor rehabilitation occurred in 652 patients (26 per cent ) and again in a higher proportion of community patients (28% vs 19%, *p* < 0.001). Total stay in “other” episodes was also longer for community patients, especially among those not transferred to rehabilitation.

### Factors which impact upon LOS

The factors which significantly affected the acute phase of hospital stay are shown in Table 3. The length of the acute phase was not significantly affected by patient age, sex or fracture type within either sub-group, but age had a minor effect in the combined population. For RAC patients the acute phase was substantially increased by surgical treatment, admission to intensive care and by complications of skin ulceration and infections - particularly in the fourteen patients with surgical site sepsis. No listed comorbid condition had any significant impact in this group.

**Table 3:** **Factors associated with acute phase LOS after hip fracture**

	**Community patients N = 1844**			**Patients from RAC N = 708**		
	Added days*	**95% CI**	***P***	**Added days**	**95% CI**	***P***
Baseline LOS**	12.4				7.3	
**Sex**	0.9	(0.1 - 1.8)	0.029		-	-
**Separation mode**			<0.001			-
Usual residence		referent			-	-
New RAC transfer	6.0	(3.6 - 8.7)				
Rehabilitation	-3.2	(-3.9, -2.5)				
Other transfer	-1.2	(-2.4, 0.0)				
Death	-2.0	(-3.3, - 0.4)			-	
**Surgery**	1.3	(0.1 - 2.5)	0.027	3.0	(1.6 - 4.6)	<0.001
**Intensive care**		-	-	4.6.	(2.3 - 7.5)	<0.001
**Comorbidities**						
Cardiac failure	2.8	(1.5 - 4.3)	<0.001		-	-
Diabetes	1.8	(0.4 - 3.4)	0.009		-	-
Stroke	2.4	(0.6 - 4.5)	0.006		-	-
**Complications**						
Delirium	2.2	(0.6 - 40)	0.006		-	-
Skin ulceration	5.4	(34 - 7.5)	<0.001	3.2	(1.4 - 5.3)	<0.001
Chest infection	3.1	(1.2 - 5.3)	0.001	1.9	(0.6 - 3.5)	0.003
Urinary infection	2.9	(1.4 - 4.4)	<0.001	2.8	(1.5 - 4.4)	<0.001
Wound infection	-	-	-	12.3	(6.0 - 21.7)	<0.001

*Mean addition to baseline value **LOS for female <80 years, cervical fracture, no surgery, rehabilitation, intensive care, comorbidity or complications; separated to usual residence.

For community patients, direct transfer to RAC extended the acute phase by six days. Cardiac failure, skin ulceration, respiratory and urinary infections were all associated with increases of at least 20 per cent of the baseline value, and diabetes, stroke and delirium by significant but lesser amounts. Community patients who died or were tranferred to rehabilitation or other units had shorter acute phases (Table 3).

The baseline value of total LOS for RAC patients was more than doubled for patients who received rehabilitation and by separation to a hospital or other facility (Table 4). Intensive care admission, and surgical site sepsis were also associated with increases exceeding 50 per cent of baseline value while increases of 20 per cent or more were associated with surgery, skin ulceration and urinary infection. Neither sex, age, fracture type nor any specific comorbidity impacted total LOS for RAC patients.

**Table 4:** **Factors associated with LOS for total hospital stay after hip fracture**

	**Community patients N = 1844**			**Patients from RAC N = 708**		
	Added days*	**95% CI**	***P***	**Added days**	**95% CI**	***P***
Baseline LOS**		14.8			**10.2**	
**Age-group**			0.001		-	
<80		referent			-	
80-84	3.5	(1.4 – 5.9)			**-**	
**85-89**	3.6	(1.6 – 5.8)			-	
90 +	2.1	(0.1 - 4.3)				-
**Fracture type**			<0.001		-	
Cervical		referent			**-**	
Unspecified	4.3	(2.7 – 6.1)			-	
Subtrochanteric	3.1	( 0.7 - 5.8)			-	
Trochanteric	2.0	(0.9 -3.1)				<0.001
**Separation mode**			<0.001			
Usual residence		referent			referent	
Other transfer	4.7	(2.8 – 6.9)		18.3	(8.8 - 32.7)	
New RAC transfer	5.1	(3.7 – 6.7)		N/A	N/A	
Death	-2.7	(-4.0, -1.3)		-0.5	(-1.9, 1.1)	<0.001
**Rehabilitation**	9.9	(8.4 - 11.4)	<0.001	13.9	(10.8 -17.4)	0.009
**Surgery**	-	-		2.4	(0.6 – 4.5)	<0.001
**Intensive care**	-	-		7.9	. (4.1 – 12.7)	
**Comorbidities**						
Parkinson’s disease	5.7	(2.3 - 9.7)	<0.001		**-**	
Diabetes	2.5	(0.9 - 4.3)	0.002		-	
Dementia	1.7	(0.0 – 3.5)	0.04		-	
**Complications**		-				
Skin ulceration	5.6	(4.0 - 7.4)		3.7	(1.7 – 5.9)	<0.001
Wound infection	4.9	(1.7 – 8.8)	<0.001	5.9	(0.9 – 13.0)	0.010
Urinary infection	2.8	(1.5 - 4.3)	0.001	4.2	(2.4 – 6.4)	<0.001
Delirium	1.9	(0.4 - 3.7)	<0.001	1.9	(0.1 – 4.1)	0.04
Chest infection	2.7	(1.0 – 4.5)	0.02	-	-	

*Mean addition to baseline value **LOS for female <80 years:cervical fracture, no surgery, rehabilitation, intensive care, comorbidity or complications; separated to usual residence.

Among community patients, those aged between 80 and 89 years had longer stay than both younger and older patients (Table 4). Patients with intracapsular fractures had shorter total stay than those with other injuries. The increase associated with rehabilitation was over 60 percent of the baseline value. Discharge to RAC or to other facilities, Parkinsonism, skin ulceration and surgical site sepsis were all associated with increases of at least 30 per cent. Community patients who died had a shorter total LOS. In the complete sample patients with dementia had a small reduction in total LOS but this was not evident within either sub-population (Table 4).

There were 763 episodes for management of conditions not coded to hip fracture or rehabilitation (652 patients). Sex, age and fracture type were not substantial determinants of LOS in this category. Diabetes, chronic respiratory disease, Parkinson’s disease, anaemia and “awaiting accommodation in another facility” (ICD10 -AM, Z751) were the most frequently identified reasons for episodes in this category (data not tabulated).

The impact of multiple LOS determinants is compound: a community patient aged 85-89 years, with subtrochanteric fracture, complications of leg ulcer and wound infection, transferred to rehabilitation and eventually discharged to RAC would have a calculated total LOS of 92 days.

## Discussion

This study has employed data linkage to identify three key findings for hospital management of hip fracture. First, the majority of hospital days (57 per cent) occurred after the acute phase, as observed in other studies [3,13]. Secondly, total LOS for patients admitted from RAC was approximately half that of those admitted from the community. Thirdly, referral to hospital-based rehabilitation effectively doubles the total LOS.

### Factors impacting LOS

The value of assessing the complete hospital experience is evident in the differing profiles of determinant factors for LOS for acute phase and total stay. Age and fracture type do not influence acute LOS but are significant factors for total stay. Transfers to other treating facilities, including rehabilitation, facilitate separation from the acute phase but result in substantially longer total LOS. Parkinsonism, diabetes and anaemia have no significant impacts on acute stay but are associated with longer total LOS. The reverse situation is seen in respect of cardiac failure. These variations are mostly due to differing rates of transfer to rehabilitation, and hospital episodes due to “other” causes. Surgery prolonged the acute phase as in English data [22], however a prolonged total stay was seen only among RAC patients.

The shorter stay for patients aged under 80 years reflected the findings of other studies [23], but unlike Scottish findings, patients aged over 90 years did not stay longer than octogenarians [12]. Fewer transfers to rehabilitation among the oldest patients was again the probable reason. Additional post-acute days and longer total stay for patients with trochanteric and subtrochanteric, compared with intracapsular fractures, reflect data from the Finnish Health Care Register [3].

It is customary in Australia for rehabilitation after hip fracture to involve transfer to a dedicated hospital unit or hospital, removed from the acute facility [23]. These transfers, while including some days of inappropriate acute care [23], still resulted in reduced LOS in acute units, as do transfers to other facilities*.* Surgery prolonged the acute phase as in English data [24], however a prolonged total stay was seen only among RAC patients. Admission into intensive care extended both acute phase and total LOS by more than 60 per cent for RAC patients but had no impact for community patients.

Of the selected comorbidities, cardiac failure and stroke in respect of acute phase and Parkinsonism and diabetes for total LOS were the only associations with substantially longer stay. The complications listed in Tables 3 and 4 were more potent in extending hospital time, particularly skin (pressure) ulceration and surgical site (wound) infections, both responsible for >30 per cent increase in acute and total LOS.

Both these conditions have been associated with considerable increases in LOS in other studies [25,26] but not examined in comprehensive mutivariate models. Systems of co-managed (orthogeriatric) care [7], resourced to promptly recognise and manage comorbidity and complications have been shown to reduce acute phase LOS, costs and the incidence of unwanted outcomes [7,8,20]. In quantifying the impact of LOS determinants at specific phases of the hospital experience, this study gives dimensions to potential benefits in both costs and reduced morbidity through timely interventions.

### Pre-fracture residence

Residential status prior to hip fracture is variously defined and variably reported [13,20,21]. Some studies exclusively address RAC patients [27], others exclude them [23,28] and many do not identify pre-fracture residence [10,24]. The findings of this study suggest that knowledge of pre-fracture residential status is vital to the understanding of the hospital trajectory for hip fracture. While other studies have previously noted a comparatively short LOS for RAC patients in the acute phase [13,20,29] and similarly for “total institutional days” [13,27], LOS in all phases of hospital stay were shorter for these patients in this study.

The difference in the acute phase was greatest (11.7 to 17.0 days, *p <*0.001) for patients who did not transfer to rehabilitation. Immediate access to post-hospital accommodation for RAC patients was the probable reason [28]. Hospital episodes for “other” reasons (comorbidities or complications) were fewer and shorter for RAC patients. Most of the difference in total LOS between RAC and community patients was attributable to the greater than threefold difference in rates of hospital-based rehabilitation. This large difference parallels findings from the Scottish Hip Fracture Audit [29].

The linkage of hospital and RAC datasets is regarded as vital to the accurate interpretation of separation codes and hence LOS data for hip fracture patients. In this study 14.4 per cent of all discharges were re-classified as transfers to RAC after examining linked data [4]. RAC patients returning to institutional care have low hospital stay [29] whereas community patients requiring new RAC placements have shown significantly longer stays than those who return to non-institutional living. Lower LOS values for patients with dementia in a large Australian study [30] possibly reflect the high proportion of institutional patients in the dementia group, who do not transfer to rehabilitation but have expedited discharges back to RAC.

### Strengths and weaknesses

Administrative databases have intrinsic strengths and weaknesses for studies of this nature. Large patient numbers and a comprehensive list of data items are major assets. Current levels of coding and transcription errors are now regarded as acceptable for meaningful analyses of diagnostic and procedural data, with reported accuracy rates as high as 96 per cent [31]. The evidence of this study suggests that separation codes in Australian hospital databases require further scrutiny [4]. The linkage facility of DVA records enabled measurement of total LOS, a wide search field for comorbidities, accurate matching of RAC status with fracture events and alignment of specific variables with components of LOS.

The principal disadvantages include the lack of information regarding disease severity, pre-fracture functional status and preoperative waiting time. Australian admission criteria make pre-fracture RAC residency at least a partial surrogate for poor functionality [17] and pre-operative delay is partly due to medical complexity [32] as reflected in comorbidity profiles.

The mean age of hip fracture patients in this study was up to 6 years greater than elsewhere reported [3,10,24]. The proportion of males was 37.6 per cent, compared with 25-30 per cent in other population-based studies [3,10,30]. These differences were reflected in a higher proportion of patients from RAC than reported from a large Scottish sample (27.7 per cent vs 21.3 per cent) [29]. DVA patients did not appear to use hospital services differently from other Australians of comparable age [4]. The distribution of fracture type was unremarkable [3,5] after 380 “unspecified or unknown” fractures (S72.00, S72.08) were proportionally reclassified.

With respect to comorbidities, this study identified higher rates for diabetes, cardiac and respiratory conditions than those drawn from the English Hospital Episode Statistics [24].

A large database study from New South Wales, Australia found dementia in 35.9 per cent of hip fracture patients aged ≥ 85 years [30]. In this study the comparable prevalence was 31.8 per cent (*p =*0.03*),* based upon less extensive data surveillance. We acknowledge that there were substantial levels of false negatives for some other key diagnoses. Targeted studies of clinical records reported delirium in between 29 and 50 per cent of hip fracture patients [33,34] and pressure ulcers in more than one third [35], both approximately three times the rates found in this study. However, comorbidity capture from linked episode data is substantially superior to that derived from acute episodes alone [36].

It is also recognised that the characteristics of individual hospitals or groupings of hospitals may contribute to differences in LOS. In this study 476 different treating hospitals were identified by code, but no information as to hospital characteristics was provided. More than half of all patients were treated in more than one hospital, with nine per cent treated in three or more hospitals. Identifying hospital-level determinants for LOS was therefore not attempted.

## Conclusion

Hip fracture patients admitted from residential care or from the community have widely different component and total LOS, for which the threefold difference in rates of transfer to rehabilitation is the major determinant. New transfer to RAC, other inter-facility transfers, Parkinsonism, pressure ulcers, and urinary and wound infections all increased LOS by at least 4 days or 25 per cent of baseline values at some phase of the hospital stay. Multiple factors associated with increased LOS had an exponential effect. These data give dimensions to potential resource efficiencies and reduced patient morbidities through targeted intervention, and emphasise the importance of specialist medical care during the acute surgical management of hip fracture patients. The additional insights provided by data linkage in studies of complex conditions are also evident.
